# Mutations of TRPM8 channels: Unraveling the molecular basis of activation by cold and ligands

**DOI:** 10.1002/med.21920

**Published:** 2022-08-17

**Authors:** Alejandro Plaza‐Cayón, Rosario González‐Muñiz, Mercedes Martín‐Martínez

**Affiliations:** ^1^ Instituto de Química Médica (IQM) CSIC Madrid Spain

**Keywords:** agonist, antagonists, mutants, structure, TRPM8

## Abstract

The cation nonselective channel TRPM8 is activated by multiple stimuli, including moderate cold and various chemical compounds (i.e., menthol and icilin [Fig. 1], among others). While research continues growing on the understanding of the physiological involvement of TRPM8 channels and their role in various pathological states, the information available on its activation mechanisms has also increased, supported by mutagenesis and structural studies. This review compiles known information on specific mutations of channel residues and their consequences on channel viability and function. Besides, the comparison of sequence of animals living in different environments, together with chimera and mutagenesis studies are helping to unravel the mechanism of adaptation to different temperatures. The results of mutagenesis studies, grouped by different channel regions, are compared with the current knowledge of TRPM8 structures obtained by cryo‐electron microscopy. Trying to make this review self‐explicative and highly informative, important residues for TRPM8 function are summarized in a figure, and mutants, deletions and chimeras are compiled in a table, including also the observed effects by different methods of activation and the corresponding references. The information provided by this review may also help in the design of new ligands for TRPM8, an interesting biological target for therapeutic intervention.

## 1 INTRODUCTION

The Transient Receptor Potential Cation Channel, Subfamily M, Member 8 (TRPM8) belongs to the Transient Receptor Potential (TRP) superfamily of ion channels and is a member of the melastatin subfamily. It has been previously known as the Cold and Menthol Receptor 1^1^ and is a nonselective ion channel, permeable to both monovalent (Na^+^, K^+^, Cs^+^) and divalent (Ca^2+^) cations, with prevalence for the latter.^2^, ^3^ TRPM8 was first identified and cloned from prostate tissue, and showed to be upregulated in prostate cancer.^4^ Shortly after, two groups cloned this channel from sensory neurons, and provide insights into its physiological functions, as a channel activated by menthol and implicated in cold perception.^1^, ^2^ Initially discovered for their role in phototransduction in *Drosophila*, TRP channels play critical roles in sensory functions, namely vision, taste, hearing, smell, touch, or osmosensation. A subgroup of TRP channels, known as thermoTRPs, has revealed crucial in thermoreception, responding to a wide range of temperatures, spanning from noxious cold to noxious heat.^5^ TRPM8 is one of these thermoTRPs, activated by cool temperatures, in mammals TRPM8 confers cold‐sensitivity from temperatures below 33°C.^6^, ^7^, ^8^, ^9^ In addition to temperature, TRPM8, as other TRPs, is a polymodal channel able to integrate several physicochemical stimuli, such as voltage, pH, osmolarity, or ligands; and these various factors interact with each other intricately modulating the gating probability of the channel.^5^ Particularly, it is well‐established that TRPM8 gating is allosterically modulated by the presence of phosphatidylinositol‐4,5‐bisphosphate (PIP_2_), a membrane phospholipid, which is required to allow ion passage,^10^, ^11^, ^12^, ^13^ and Ca^2+^, which can downregulate the channel following activation, through two temporally different mechanism, acute desensitization, and tachyphylaxis.^14^ Acute desensitization is dependent on PIP_2_ availability and Ca^2+^‐Calmodulin, whereas tachyphylaxis depends on PIP_2_ hydrolysis, protein kinase C, and phosphatases.^14^ Furthermore, TRPM8 has shown gating modulation by multiple exogenous chemicals, both agonists, which can activate the channel in absence of cold, and antagonists, that block the channel preventing gating.^15^, ^16^, ^17^

The gating dynamics rely on an elaborate regulatory network that modulates TRPM8 activity by a wide range of mechanisms, including differential transcription and alternative splicing, vesicle trafficking to the membrane,^18^ posttranslational modifications,^19^, ^20^, ^21^, ^22^ and the involvement of several regulatory proteins.^23^, ^24^, ^25^ Both this complex regulation and polymodal gating of TRPM8, along with the expression in multiple tissues, strongly suggest additional physiological roles for the channel besides thermoception. This is further supported by several variants in the *TRPM8* gene being associated to a broad spectrum of pathologies.^26^, ^27^, ^28^, ^29^, ^30^

The mechanism underlying cold detection, the most established role of TRPM8, involves expression of the channel in peripheral afferent neurons (Aδ and C fibers) innervating the skin, in which, upon cold detection, TRPM8 channels undergo conformational changes that lower their voltage threshold of activation, reaching physiological potentials, allowing gating and the consequent influx of cations which leads to cell depolarization.^31^, ^32^ The cold‐evoked depolarizing receptor potential generated at the nerve endings travels as a wave of action potential firing to the neuron body, and then travels up to the brain, where the subjective feeling of cold is generated, as well as behavioral and autonomic responses to avoid heat loss.^33^ In a similar way, agonists, such as menthol, are also able to shift the voltage‐activation curves toward more negative potentials, and thus allow the opening of the channel at physiological membrane potentials. On the contrary, antagonists shift the curve toward more positive potentials.^32^, ^34^

It is well‐established that the cold‐signaling pathway is capable of inhibit pain signaling and, accordingly, TRPM8 activation by agonists exerts analgesic effects.^35^ In this sense, menthol, a TRPM8 agonist, has been used for pain relief for many years and is included in several commercial topical analgesics indicated for muscular and articular pain. TRPM8 channels are also implicated in cold hyperalgesia and cold allodynia, and therapies involving TRPM8 modulation may ameliorate these conditions.^36^ Moreover, some genome‐wide association studies have found some single‐nucleotide polymorphisms (SNPs) located in the *TRPM8* gene that have been associated with migraine.^37^ Furthermore, studies in preclinical models suggest a contribution of this ion channel to migraine, although there are several aspects that need to be addressed to confirm this point.^37^

TRPM8 is also expressed in a variety of tissues not exposed to the environment, which supports that they may have additional physiological roles. One of them is the brown adipose tissue, which is involved in energy expenditure. It has been shown that TRPM8 activation increases thermogenesis, and might prevent obesity and abnormal glucose homeostasis.^38^ The vasculature is another tissue in which TRPM8 is present. Preliminary studies of some cardiovascular disorders, including hypertension or some cold‐triggered responses as Reynaud's phenomenon, suggest patients may benefit from TRPM8‐targeted therapies.^39^, ^40^, ^41^ TRPM8 is also found in corneal afferents fibers, where it plays a crucial role in detection of osmolality in the eye, where increases in osmolality seem to shift TRPM8 activation threshold toward physiologically relevant temperatures, thus allowing activation and signaling, in a process which appears to control the blink rate.^42^ Consequently, dry eye discomfort symptoms may benefit from modulation of the blink rate and tear secretion with TRPM8 agonists.^43^ The epithelial cells or visceral afferents that innervate the gastrointestinal tract and airways are other tissues in which TRPM8 channels have been identified. In the intestinal tract, TRPM8 might contribute to inflammatory diseases, such as irritable bowel syndrome^44^ or colitis.^45^, ^46^ Further, TRPM8 has been implicated in some urinary bladder dysfunctions.^47^ TRPM8 seems overexpressed in patients with painful bladder syndrome and idiopathic detrusor overactivity, where the levels of TRPM8 appear to correlate with the perceived pain and frequency of micturition.^48^ Therefore, modulation of TRPM8 may alleviate the symptomatology of these bladder dysfunctions and pathologies, as it has been shown in animal models.^49^

TRPM8 channels have also been associated with different types of cancer, where they seem to contribute to cellular proliferation and migration. In some cases, especially in highly steroid‐dependent tissues, as prostate or breast, the relationship between TRPM8 and signaling by sex steroid hormones might be the basis of these associations.^50^, ^51^ Along similar lines, sex differences in cold perception^52^ and subsequent cold adaptation responses^53^ appear to be due to differential sex hormones regulation. Overexpression of TRPM8 in prostate cancer cells might position *TRPM8* mRNA levels as a promising diagnostic marker, as they have shown diagnostic advantages in comparison to other biomarkers.^54^ Additionally, TRPM8 Ca^2+^ signaling appears to be essential for the physiopathology of prostate cancer.^55^ In the case of breast cancer, overexpression of TRPM8 has also been reported, which appears to be regulated by estrogen receptor alpha,^50^ and its expression levels are strongly correlated to proliferative parameters,^56^ hence making TRPM8 modulation strategies promising treatment alternatives.

Although the channel existence and thermoceptive function had been known for years, the tridimensional (3D) structure of the channel was first resolved in 2017, from the bird *Ficedula albicollis* (Collared flycatcher, *fa*TRPM8), using cryo‐electron microscopy (cryo‐EM).^57^ Since then, seven additional structures of TRPM8 have been resolved, either from *F. albicollis* or from *Parus major* (Great tit, *pm*TRPM8), both in a ligand‐free state or bound to several ligands (agonists: icilin and WS‐12; antagonists: AMTB, and TC‐I 2014, Figure 1), and with or without allosteric modulators PIP_2_ and Ca^2+^.^13^, ^58^ It is of interest to point out that, through the manuscript, we will use the *pm*TRPM8 cryo‐EM residue numbering, which differs in four residues from the numbering in UniProt sequence A0A5S8WF66‐1,^59^ as shown in the SI alignment. As other thermoTRPs structures, and in accordance with previous predictions from the amino acid sequence, TRPM8 is a homotetramer, with each protomer comprising a transmembrane domain and cytoplasmatic N‐ and C‐termini.

**Figure 1:**
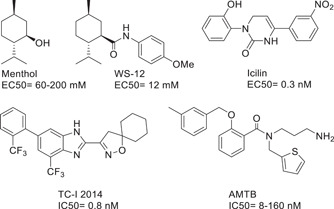
Menthol and TRPM8 agonists and antagonists present in cryo‐EM structures of TRPM8^13^, ^58^

**Figure 2:**
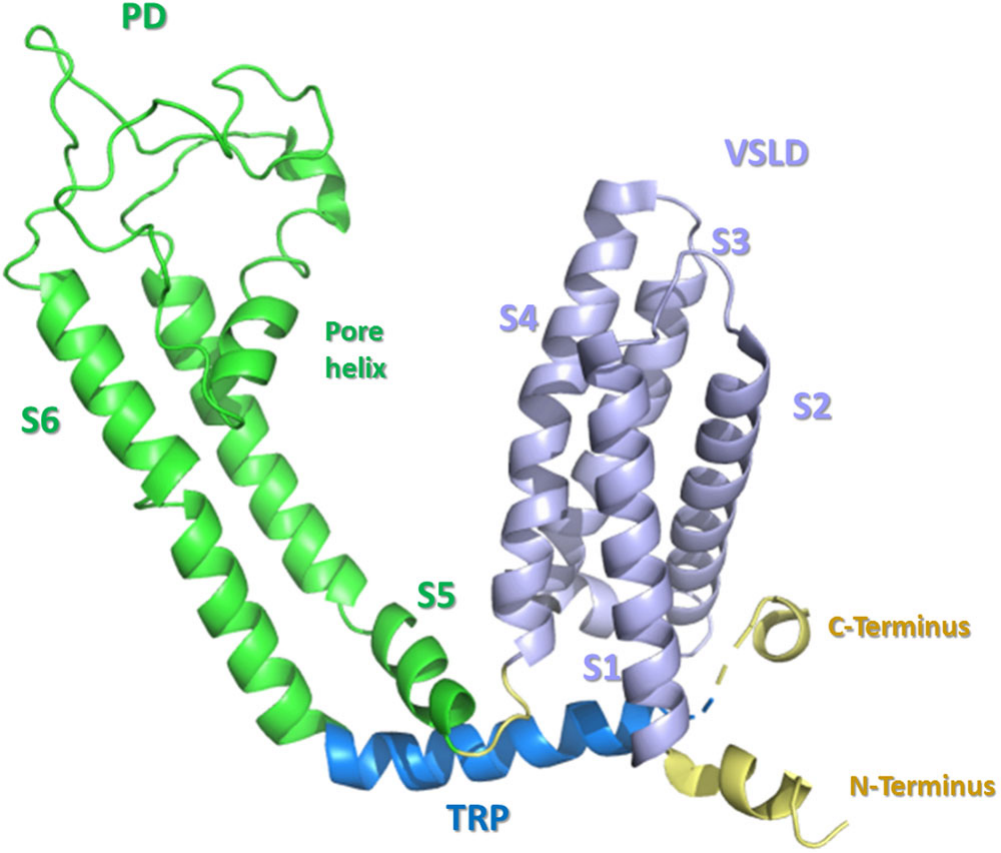
Schematic representation of the transmembrane region of TRPM8 (extracted from PDB ID 6O77 cryo‐EM structure)^58^ [Color figure can be viewed at wileyonlinelibrary.com]

The transmembrane region comprises a voltage‐sensor‐like domain (VSLD) with four transmembrane α‐helices (S1 to S4), a pore domain (PD) with two additional transmembrane α‐helices (S5 and S6), and a pore helix (Figure 2). The assembly of the complete tetramer involves the interaction of the PD of one protomer with the VSLD of the adjacent protomer, thus forming a domain‐swapped tetramer.^57^

In the cytosolic N‐terminal region, TRPM8 shares with every other member of the TRPM family a large segment containing 4 high homology regions (*Melastatin Homology Regions*, MHRs), which appear to contribute to the correct assembly and function of the tetramer. The pre‐MHR region (approximately the first 60 residues) has shown a pivotal role in biogenesis, trafficking to membrane, and activity of the channel.^60^, ^61^ Recently, the N‐terminal region of TRPM channels has been associated with a nucleotide binding domain (the so‐called SLOG domain).^62^ The cytosolic C‐terminus, on the other hand, contains the *Transient Receptor Potential* (TRP) domain, an α‐helical region of 25 residues, moderately conserved along TRP channels. In TRPM8 channels, the TRP domain is located beneath the VSLD, where it delimits the lower part of the binding pocket, and exerts an essential role in TRPM8 function and response to exogenous ligands. This is followed by two cytosolic α‐helices, and a final terminal helix that conforms a coiled coil domain with the corresponding helices of the other protomers.^13^, ^58^

The known 3D structures of the TRPM8 channel along with mutagenesis studies have allowed gaining knowledge into its gating mechanisms and regulation, including responses to a variety of stimuli, such as temperature, voltage, ligands, and lipids. In this review, we will discuss the key TRPM8 residues described so far, which participate in the modulation of the response to cold and/or ligands. Moreover, this information will be combined with that derived from the 3D structures of TRPM8, in free or ligand bound states. The interplay between mutagenesis and structural data is of interest to unravel TRPM8 channels mechanisms, and could aid in the design of new ligands. Furthermore, comparison among different species provides insights on TRPM8 residues that have changed over the course of evolution, depending on environmental conditions.

## 2 TRPM8 MUTANTS

**Figure 3:**
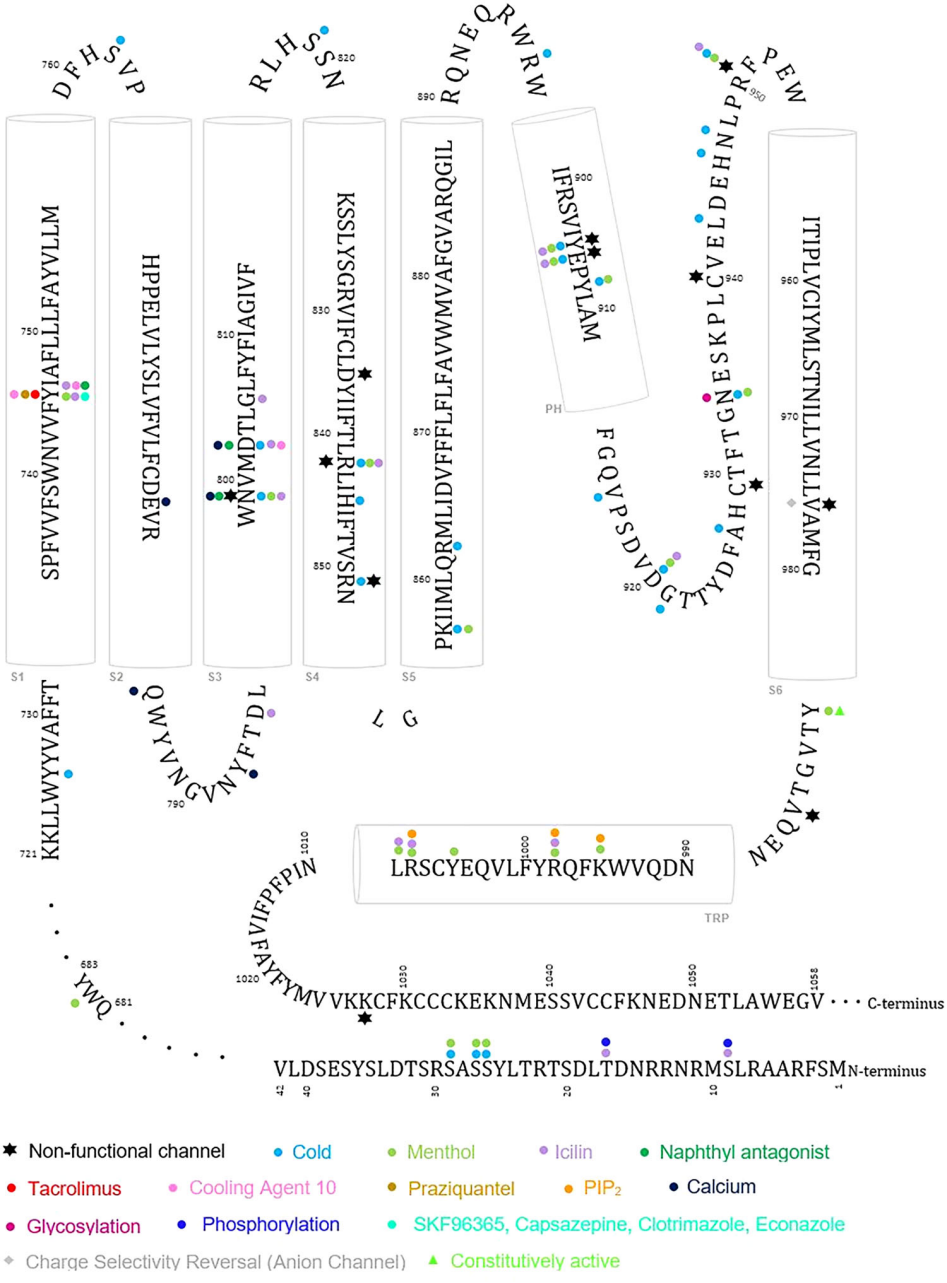
Summary of important residues in TRPM8 function identified in mutagenesis studies. Upon mutation of a given residue, colored circles indicate disrupted processes as shown by legend. The *hs*TRPM8 sequence has been used to integrate information from every studied species. Cylinders indicated α‐helices based on the secondary structure analysis of the different structures deposited in the Protein Data Bank [Color figure can be viewed at wileyonlinelibrary.com]

To get insights into TRPM8 channels modulation by cold or ligands, several mutagenesis studies have been carried out. Different groups have generated a variety of mutants involving residues that are spread all over the channel, with a greater number within the transmembrane region (TM) (Figure 3, Table 1). Within this region, the analysis of TRPM8 sequences from different species identified the S4 and S5 transmembrane segments as highly conserved.^92^ The *hs*TRPM8 VSLD, spanning from S733 to N852 (S1–S4), together with the TRP domain (N990 to R1008) have been broadly studied, as they are considered the binding zone of exogenous compounds. Even before the resolution of cryo‐EM TRPM8 structures, it had already been postulated that menthol binds to the VSLD.^72^

**Table 1:** Summary of TRPM8 mutations, chimeric constructs and deletions, and their effect upon cold and ligand stimuli

Mutation/chimeras/deletions	Stimuli/PTM	Effect on the mutant channel	Species	References
S9A	Icilin	Insensitive	*hs*TRPM8	[63]
	Phosphorylation	Impaired phosphorylation	*hs*TRPM8	[63]
T17A	Icilin	Insensitive	*hs*TRPM8	[63]
	Phosphorylation	Impaired phosphorylation	*hs*TRPM8	[63]
S26D	Cold/menthol	WT phenotype	*mm*TRPM8	[61]
S26P	Cold/menthol	Hypersensitive phenotype	*mm*TRPM8	[61]
S26V	Cold/menthol	WT phenotype	*mm*TRPM8	[61]
S27A	Cold/menthol	WT phenotype	*mm*TRPM8	[61]
S27D	Cold/menthol	WT phenotype	*mm*TRPM8	[61]
S27P	Cold/menthol	Hypersensitive phenotype	*mm*TRPM8	[61]
S29A	Cold/menthol	Enhanced response	*mm*TRPM8	[64]
R30Q	Menthol	Enhanced response	*hs*TRPM8	[65]
G150R	WS‐12	Undetectable response	*hs*TRPM8	[66]
K423N	WS‐12	Reduced responses	*hs*TRPM8	[66]
R475C	WS‐12	Reduced responses	*hs*TRPM8	[66]
R485W	WS‐12	Undetectable response	*hs*TRPM8	[66]
Y516E	WS‐12	Undetectable response	*hs*TRPM8	[66]
W682A	Menthol	No response	*rnTRPM8*	[67]
Y745A	Menthol	Insensitive	*mm*TRPM8	[68]
	Icilin/cooling agent 10	Insensitive	*hs*TRPM8	[69]
Y745F	Menthol	Reduced potency	*mm*TRPM8	[68]
Y745H	Cold	WT responses	*mm*TRPM8	[68, 70, 71]
	Menthol	Insensitive, no cold response potentiation	*mm*TRPM8	[68, 70]
	Menthol	No significant differences in binding	*hs*TRPM8‐VSLD	[72]
	Menthol	Impaired binding	*hs*TRPM8	[73]
	Icilin	Insensitive, no cold response potentiation	*mm*TRPM8	[68]
	Tacrolimus	WT‐like activation, no cold responses potentiation	*mm*TRPM8	[74]
	Praziquantel	No agonist activity, reduced cold responses potentiation	*hs*TRPM8	[71]
	BCTC	WT‐like inhibitory responses	*mm*TRPM8	[70]
	SKF96365	No inhibition	*mm*TRPM8	[70]
	Capsazepine Clotrimazole Econazole	Reduced inhibition	*mm*TRPM8	[70]
	PIP_2_	WT affinity	*mm*TRPM8	[68]
I746A	Icilin/cooling agent 10	Reduced efficacy	*hs*TRPM8	[69]
	Naphthyl antagonist	Reduced inhibition	*hs*TRPM8	[69]
L774ANAP	Menthol	ANAP peak emission redshifted	*mm*TRPM8	[75]
V791ANAP	Menthol	ANAP peak emission redshifted	*mm*TRPM8	[75]
D796R	Cold/menthol	Sensitive	*hs*TRPM8	[76]
	Icilin	No response, attenuates response to menthol	*hs*TRPM8	[76]
	Glycosylation	WT phenotype	*hs*TRPM8	[76]
Q785A	Menthol	Dismissed desensitization	*pmTRPM8*	[58]
N790A	Menthol	Dismished desensitization	*pmTRPM8*	[58]
N799A	Cold/menthol	Sensitive	*rn*TRPM8	[77]
	Icilin	Insensitive, maintaining cold responses potentiation	*rn*TRPM8	[77]
	Icilin	Insensitive	*hs*TRPM8	[69]
	Cooling Agent 10	Sensitive	*hs*TRPM8	[69]
	Tacrolimus	WT phenotype	*mm*TRPM8	[74]
	Naphthyl antagonist	Enhanced potency	*hs*TRPM8	[69]
N799D	Cold/menthol	Reduced responses	*hs*TRPM8	[78]
	Icilin	No response	*hs*TRPM8	[78]
	Glycosylation	No matured *N*‐glycosylated isoform	*hs*TRPM8	[78]
N799E	Icilin	Insensitive	*rn*TRPM8	[77]
N799Y	Icilin	Insensitive	*rn*TRPM8	[77]
D802A	Cold/menthol	Sensitive	*rn*TRPM8	[77]
	Icilin	Insensitive	*hs*TRPM8	[69]
	Icilin	Insensitive, no cold response potentiation	*rn*TRPM8	[77]
	Cooling Agent 10	Reduced potency	*hs*TRPM8	[69]
	Camphor	WT phenotype	*hs*TRPM8	[79]
	Naphthyl antagonist	Reduced potency	*hs*TRPM8	[69]
D802E	Cold/menthol	WT responses	*rn*TRPM8	[77]
	Icilin	Insensitive	*rn*TRPM8	[77]
D802H	Cold/menthol	WT responses	*rn*TRPM8	[77]
	Icilin	Insensitive	*rn*TRPM8	[77]
D802K	Cold/menthol	WT responses	*rn*TRPM8	[77]
	Icilin	Insensitive	*rn*TRPM8	[77]
D802N	Cold	Reduced responses	*hs*TRPM8	[78]
	Cold/menthol	WT responses	*rn*TRPM8	[77]
	Cold/menthol	Sensitive	*hs*TRPM8	[76]
	Menthol	Increased current	*hs*TRPM8	[78]
	Icilin	Insensitive	*hs*TRPM8	[76, 78]
	Icilin	Insensitive	*rn*TRPM8	[77]
	Glycosylation	WT phenotype	*hs*TRPM8	[76]
D802Q	Cold/menthol	WT responses	*rn*TRPM8	[77]
	Icilin	Insensitive	*rn*TRPM8	[77]
D802R	Cold/menthol	Almost WT currents	*hs*TRPM8	[76]
	Icilin	Insensitive	*hs*TRPM8	[76]
	Glycosylation	WT phenotype	*hs*TRPM8	[76]
D802S	Cold/menthol	WT responses	*rn*TRPM8	[77]
	Icilin	Insensitive	*rn*TRPM8	[77]
D802Y	Cold/menthol	WT responses	*rn*TRPM8	[77]
	Icilin	Insensitive	*rn*TRPM8	[77]
A805	Icilin	Insensitive	Naturally present in the avian TRPM8	[77]
G805A	Cold/menthol	WT responses	*rn*TRPM8	[77]
	Icilin	Insensitive, no cold response potentiation	*rn*TRPM8	[77]
	Praziquantel	WT responses	*hs*TRPM8	[71]
S824ANAP	Menthol	ANAP emission redshifted	*mm*TRPM8	[75]
D835R	Any	Nonfunctional channel	*hs*TRPM8	[76]
	Glycosylation	No matured *N*‐glycosylated isoform	*hs*TRPM8	[76]
R842A	Cold	Reduced responses	*hs*TRPM8	[73]
	Menthol	Reduce response	*hs*TRPM8	[73]
R842D/E	Cold/menthol/icilin	No response	*hs*TRPM8	[76]
	Glycosylation	No matured *N*‐glycosylated isoform	*hs*TRPM8	[76]
R842H	Menthol	Reduce response	*hs*TRPM8	[73]
	Menthol	Similar affinity as WT	*hs*TRPM8‐VSLD	[72]
R842K	Menthol	Reduced responses	*hs*TRPM8	[73]
H844A	Menthol	WT response	*faTRPM8*	[13]
	Icilin	Strongly decrease response	*faTRPM8*	[13]
H845A	Cold	Reduced responses	*hs*TRPM8	[73]
	Menthol	WT responses	*hs*TRPM8	[73]
R851A	Any	Nonfunctional channel	*hs*TRPM8	[73]
R851Q	Cold	Reduced responses	*hs*TRPM8	[73]
	Menthol	WT responses	*hs*TRPM8	[73]
K856A	Cold	Hypersensitive phenotype	*hs*TRPM8	[73]
	Menthol	Enhanced sensitivity and affinity	*hs*TRPM8	[73]
K856R	Menthol	WT responses	*hs*TRPM8	[73]
R862A	Cold	Reduced responses	*hs*TRPM8	[73]
	Menthol	WT responses	*hs*TRPM8	[73]
A875ANAP	Menthol	ANAP emission redshifted	*mm*TRPM8	[75]
R897E	Cold	Reduced responses	*mm*TRPM8	[80]
	Menthol	WT responses	*mm*TRPM8	[80]
Y905A	Cold/menthol/icilin	Nonfunctional	TRPM8^a^	[81]
Y905F	Cold/menthol/icilin	WT responses	TRPM8^a^	[81]
Y905W	Cold/menthol/icilin	Strongly decreased current	TRPM8^a^	[81]
E906A	Cold/menthol/icilin	Inactive channel	TRPM8^a^	[81]
E906Q	Cold/menthol/icilin	Reduced responses	TRPM8^a^	[81]
Y908A/W	Cold/menthol	Strongly decreased current	TRPM8^a^	[81]
	Icilin	WT responses	TRPM8^a^	[81]
Y908F	Cold/menthol/icilin	WT responses	TRPM8^a^	[81]
D918A/E/N	Cold/menthol/icilin	WT responses	TRPM8^a^	[81]
Y919V^b^	Cold	Increased current	*af*TRPM8	[82]
V919I	Cold/menthol/icilin	WT responses	TRPM8^a^	[81]
V919Y	Cold	Decreased current	*la*TRPM8	[82]
D920A/N	Cold/menthol/icilin	Drastic decrease in currents	TRPM8^a^	[81]
D920ANAP	Menthol	ANAP emission redshifted	*mmTRPM8*	[75]
T922ANAP	Menthol	ANAP emission blueshifted	*mmTRPM8*	[75]
G925I	Cold	Enhanced sensitivity	*la*TRPM8	[82]
G925Q	Cold	Decreased sensitivity	*la*TRPM8	[82]
G925ANAP	Cold	ANAP emission redshifted, enhanced cold sensitivity	*la*TRPM8	[82]
C929A	Menthol/icilin/cold	Nonfunctional channel	*mm*TRPM8	[83]
N934D	Cold	Shifted threshold to lower temperature. Reduced responses	*mm*TRPM8	[20]
	Menthol	Reduced potency	*mm*TRPM8	[20]
N934K	Cold	Shifted threshold to lower temperature. Reduced responses	*mm*TRPM8	[20]
	Menthol	Reduced potency	*mm*TRPM8	[20]
N934Q	Cold	Shifted threshold to lower temperature. Reduced responses	*mm*TRPM8	[20]
	Cold	WT‐like threshold, decreased current	*mm*TRPM8	[83]
	Menthol	Reduced responses	*mm*TRPM8	[20, 84]
	Menthol/icilin	WT responses	*mm*TRPM8	[83]
	Glycosylation	No glycosylation	*mm*TRPM8	[20, 83]
L939ANAP	Menthol	ANAP emission redshifted	*mmTRPM8*	[75]
C940A	Menthol/icilin/cold	Nonfunctional channel	*mm*TRPM8	[83]
C940G/R	Cold/menthol/icilin	Absence of current	TRPM8^a^	[81]
L943K	Cold	Decreased sensitivity	*la*TRPM8	[82]
L943ANAP	Cold	ANAP emission redshifted, enhanced cold sensitivity	*la*TRPM8	[82]
L947Q	Cold	Decreased sensitivity	*la*TRPM8	[82]
L947ANAP	Cold	ANAP emission redshifted, enhanced cold sensitivity	*la*TRPM8	[82]
R950E	Cold/menthol/icilin	No current	TRPM8^a^	[81]
P958ANAP	Menthol	ANAP emission redshifted	*mmTRPM8*	[75]
S966ANAP	Menthol	ANAP emission redshifted	*mmTRPM8*	[75]
V976D	Cold/menthol/icilin	Nonconducting	*rn*TRPM8	[85]
V976K		Anion channel	TRPM8^a^	[86]
Y981E/K		Constitutively active channel	*rn*TRPM8	[85]
Y981F	Menthol	No response	*rn*TRPM8	[85]
Y981L	Menthol	Decreased efficacy	*rn*TRPM8	[85]
V986A/G/F		Nonfunctional channels	*rn*TRPM8	[85]
K995Q	Icilin	WT phenotype	*rn*TRPM8	[12]
	PIP_2_	Decreased sensitivity to PIP_2_, higher susceptibility to PIP_2_ depletion	rnTRPM8	[12]
R998Q	Menthol	No response	*rdTRPM8*	[67]
	Icilin	Insensitive	*rn*TRPM8	[12]
	PIP_2_	Decreased sensitivity to PIP2, higher susceptibility to PIP_2_ depletion	*rn*TRPM8	[12]
Y1005A	Cold	WT response	*mm*TRPM8	[68]
	Menthol	Reduced responses	*mm*TRPM8	[68]
Y1005F	Cold	Reduced responses	*mm*TRPM8	[68]
	Menthol	Reduced responses	*mm*TRPM8	[68]
R1008Q	Menthol	Decreased sensitivity	*rn*TRPM8	[12]
	Icilin	Insensitive	*rn*TRPM8	[12]
	PIP_2_	Decreased sensitivity to PIP_2_, higher susceptibility to PIP_2_ depletion	*rn*TRPM8	[12]
L1009A	Menthol	WT responses	*mm*TRPM8	[68]
L1009P	Menthol	WT responses	*mm*TRPM8	[68]
L1009R	Cold	WT response profiles, larger responses	*mm*TRPM8	[68]
	Menthol	Significantly reduced responses, induced cold responses potentiation at high concentration	*mm*TRPM8	[68]
	Menthol	WT [^3^H]‐menthol binding	*hs*TRPM8	[73]
	Icilin	No response, no cold response potentiation	*mm*TRPM8	[68]
K1026Q	PIP_2_	No decrease in current	*rn*TRPM8	[12]
K1027Q		Nonfunctional channel	*rn*TRPM8	[12]
K1030Q	PIP_2_	No decrease in current	*rn*TRPM8	[12]
K1034Q	PIP_2_	No decrease in current	*rn*TRPM8	[12]
K1036Q	PIP_2_	No decrease in current	*rn*TRPM8	[12]
L1089P		No functional channel	*h*TRPM8	[84]
R52K + F56Y + T58V + R59E	Cold + menthol	Reduced responses	*mmT*RPM8	[61]
	Glycosylation	No matured *N*‐glycosylated isoform	*mmT*RPM8	[61]
T58V + R59E	Cold + menthol	Reduced responses (defects in channel biogenesis and trafficking)	*mmT*RPM8	[61]
	Glycosylation	No matured *N*‐glycosylated isoform	*mmT*RPM8	[61]
H726Y + A762S + P819S + A927S + H946Y + S947N	Cold	Enhanced sensitivity	*it*TRPM8	[87]
	Icilin	WT response	*it*TRPM8	[87]
S762E + V763L	Menthol WS‐12 AMTB	Sensitive	*hs*TRPM8	[88]
D796R + R842D	Any	No response	*hs*TRPM8	[76]
	Glycosylation	No matured *N*‐glycosylated isoform	*hs*TRPM8	[76]
N799D + D802N	Cold	Reduced responses	*hs*TRPM8	[78]
	Menthol	Increased currents	*hs*TRPM8	[78]
	Icilin	Insensitive	*hs*TRPM8	[78]
V800K + M801L	Cold/menthol	Strong decreased sensitivity	*hs*TRPM8	[78]
	Glycosylation	No matured *N*‐glycosylated isoform	*hs*TRPM8	[78]
D802R + D835R	Any	Nonfunctional channel	*hs*TRPM8	[76]
	Glycosylation	No matured *N*‐glycosylated isoform	*hs*TRPM8	[76]
D802N + R842D	Menthol	Reduced responses	*hs*TRPM8	[76]
D802R + R842D	Cold	Reduced responses	*hs*TRPM8	[76]
	Menthol	Reduced responses	*hs*TRPM8	[76]
	Icilin	Insensitive	*hs*TRPM8	[76]
	Glycosylation	WT phenotype	*hs*TRPM8	[76]
D802R + R842E	Cold/menthol	Reduced responses	*hs*TRPM8	[76]
	Icilin	Insensitive	*hs*TRPM8	[76]
	Glycosylation	WT phenotype	*hs*TRPM8	[76]
F839R + H845R or F839R + H845R + T848K	Menthol	WT responses	*hs*TRPM8	[76]
	Icilin	Insensitive	*hs*TRPM8	[76]
D918A + D920A	Cold/menthol/icilin	Strongly decreased current	TRPM8	[81]
D918N + D920N	Cold/menthol/icilin	Inactive	TRPM8	[81]
C929A + C940A	Menthol/icilin	Desensitization	*mm*TRPM8	[83]
F1045A + K1046G	Menthol/WS‐12/AMTB	Sensitive	*hs*TRPM8	[88]
^507–556^‐*gg*TRPM8 + *mm*TRPM8 chimera	Cold/menthol	Increase response	*mm*TRPM8‐*gg*TRPM8	[80]
^507–546^‐*gg*TRPM8 + *mm*TRPM8 chimera	Cold	WT responses	*mm*TRPM8‐*gg*TRPM8	[80]
	Menthol	Increase response	*mm*TRPM8‐*gg*TRPM8	[80]
^546–556^‐*gg*TRPM8 + *mm*TRPM8 chimera	Cold	Increase response	*mm*TRPM8‐*gg*TRPM8	[80]
	Menthol	Moderately increase response	*mm*TRPM8‐*gg*TRPM8	[80]
^803^VGAILL^808^‐TRPM8	Menthol/WS‐12/CPS‐154/CPS‐369	WT phenotype	*hs*TRPM8	[89]
	Icilin	Insensitive	*hs*TRPM8	[89]
^848^AIHKQ^852^‐TRPM8	Cold	WT responses	*hs*TRPM8	[73]
	Menthol	Decreased sensitivity, and reduced [^3^H]‐menthol binding	*hs*TRPM8	[73]
^980^GETVNK^985^‐TRPM8		Constitutively active channel	*rnTRPM8*	[85]
^980^GETVNK^985^‐TRPM8 + V986I or + N989E or + N990S		Prevent constitutive activity of ^980^GETVNK^985^‐TRPM8	*rnTRPM8*	[85]
^980^GETVNKIAQES^990^‐TRPM8		Prevent constitutive activity of ^980^GETVNK^985^‐	*rnTRPM8*	[85]
^1009^PAA^1011^‐TRPM8	Menthol (and isomers)	Altered stereoselectivity	*mm*TRPM8	[68]
	Icilin	Reduced responses, maintaining cold response potentiation	*mm*TRPM8	[68]
TRPV1 C_t_ + TRPM8 chimera	Temperature	TRPV1‐like responses	*rn*TRPM8	[90]
	Menthol	WT responses	*rn*TRPM8	[90]
	Capsaicin	Insensitive	*rn*TRPM8	[90]
	PIP_2_	Inhibitory response	*rn*TRPM8	[90]
TRPM8‐ΔN_t_39	Cold/menthol	WT responses	*rnTRPM8*	[60]
TRPM8‐ΔN_t_86	Cold/menthol	No response	*rnTRPM8*	[60]
TRPM8‐ΔN_t_116	Cold/menthol	No response	*rnTRPM8*	[60]
TRPM8‐ΔN_t_245	Cold/menthol	No response	*rnTRPM8*	[60]
TRPM8‐ΔN_t_352	Cold/menthol	No response	*rnTRPM8*	[60]
TRPM8‐ ΔC_t_36	Temperature	Decreased sensitivity	*rn*TRPM8	[91]
TPRM8‐∆C1070	Cold/menthol	No response	*rnTRPM8*	[60]

Abbreviations: PTM, posttranslational modifications; WT, wild‐type.

^a^ Species not indicated in the publication.

^b^ afTRPM8 Y914 in our alignment Supporting Information: Figure S2, sequence retrieved from UniProt database.

### 2.1 N‐terminal domain

To analyze the role of the N‐terminal region in TRPM8, different mutant, deletion, and chimeric constructs have been studied. C. Phelps and R. Gaudet generated five TRPM8 deletion mutants, ∆N39, ∆N86, ∆N116, ∆245, and ∆352, the two latter lacking either the first, or the first and the second MHRs.^60^ With the exception of ∆N39 TRPM8, which displayed WT‐like responses, there was no response to menthol or cold for the other deletion mutants, besides, they were not present in the plasma membrane. Pertusa et al.^61^ also performed mutagenesis and chimeric constructs studies in an attempt to further understand the role of this region. This study showed that the start of the N‐terminal domain plays important roles in biogenesis and function of the TRPM8 channels. In particular, through a series of deletions, chimeras and point mutant constructs, a segment encompassing residues 40–60 was identified as crucial in channel folding and assembly. Within this sequence, the chimera in which residues 50–59 had been replaced by the corresponding ones in TRPM2 displayed a novel phenotype. In this segment, TRPM8 and TRPM2 differ in four residues, R52(K), F56(Y), T58(V), and R59(E). This chimera is mainly localized in the endoplasmic reticulum (ER) and no mature glycosylation was observed, even though 50% of the transfected cells had a detectable response to cold and menthol when applied simultaneously. Most of this phenotype was due to alterations in residues T58 and R59, as the *mm*TRPM8 double mutant **T58V** + **R59E** showed impaired biogenesis and channel trafficking to plasmatic membrane, absence of the mature *N*‐glycosylated isoform, as well as defects in its responses to cold and menthol (showing only reduced responses after simultaneous application of both stimuli). Based on these results, a TRPM8 splice variant, found in prostate cancer cells and lacking the first 65 residues, was also studied.^61^, ^93^, ^94^ Thus, the truncated *hs*TRPM8 that mimics this splice variant is mostly retained in the ER, and is not able to respond to cold and menthol. Additionally, deletions or substitutions of the initial segment spanning from residues 1–40 yielded functional channels and induced enhanced sensibility to cold and menthol.^61^ Alterations in the segment spanning from residues 10–20 led to a decrease in channel sensitivity. Accordingly, the group of N. Prevarskaya has shown that **S9A** and **T17A** *hs*TRPM8 mutants yield channels less sensitive to icilin due to faulty posttranslational phosphorylation.^63^ Conversely, residues 20–30 are responsible for a hypersensitive phenotype.^61^ Further characterization of this segment led to the identification of S26 and S27 as the residues involved in this mechanism. Considering that only their mutations to proline (**S26P** or **S27P**), but not to other amino acids (**S26V**, **S26D**, **S27A**, and **S27D**), were able to replicate the hypersensitive behavior, it was thought not to be due to posttranslational modifications, but rather to conformational changes involving the Pro residue. The point mutation **S27P** reduced EC_50_ values of menthol by almost half of WT‐*mm*TRPM8, and showed a marked increase in current amplitudes in response to menthol or cold. This behavior could be attributed to a leftward shift of the voltage dependence of activation, thus, leading to a higher channel opening probability at physiological membrane potentials. In a recent study, the same authors identified S26 as a phosphorylation site, together with S29, S541, and S542, and showed that there is a TRPM8 downregulation by basal phosphorylation.^64^ Point mutations to Ala in each of these Ser, showed that S29 is a key residue for TRPM8 modulation, as only the **S29A** mutation led to a strong potentiation of the response to cold and menthol. Besides, **S29A** mutant showed a decrease of functional channels at plasma membrane. Considering the role of Ser phosphorylation as a negative TRPM8 modulator, and the importance of conformational flexibility to facilitate phosphorylation, mutant **S27P** has been suggested to modify the N‐terminal region, preventing its negative modulation. Recently, the **R30Q** TRPM8 variant has been studied, one of the mutant channels present in patients with trigeminal neuralgia.^65^ This is also a gain‐of‐function mutation, with a left‐shift of the voltage curve. Additionally, an increase in the response to menthol in **R30Q** TRPM8 compared to WT was also observed. Based on previous studies on S26 and S27 it was argued that this mutation might influence the stability of the TRPM8 *N*‐terminus. The identification of the key role of S29 led to the hypothesis that this **R30Q** might prevent S29 phosphorylation or the effect of this posttranscriptional modification.^64^

The N‐terminus was also explored by Morgan et al.^66^ that analyzed the effect of nonconserved substitutions located within conserved regions, namely TRPM8 rare variants **G150R**, **K423N**, **R475C**, **R485W**, and the experimental mutant **Y516E**. The rare variants were selected from the human genome SNP database, and are rather infrequent in human population. In response to agonist WS‐12, only **K423N** and **R475C** showed activation, although significantly reduced compared to WT‐TRPM8, and only at substantially high WS‐12 concentrations. The authors indicated that further studies were necessary to understand the reason for this behavior, although they suggested problems in the generation of a fully functional channel, or poor expression levels.

Pertusa et al.^80^ explored chimeras of the N‐terminal region, replacing *mm*TRPM8 by the homologous sequence from the chicken channel. The 507–556 region was important for showing an increased response to cold and menthol. This chimera produced a shift in the voltage dependence curve toward more negative potentials, and an increase in maximal conductance. As there was not an increase in expression levels, this behavior might be related to this region being involved in a higher recruitment of TRPM8 channels in the plasma membrane. In this 50 amino acids sequence, the first 20 residues are highly conserved, therefore, two chimeras, 507–546 and 546–556, were designed. Both chimeras displayed an increase in menthol response, although barely moderate for the latter, whereas only the 546–556 showed an increase in cold‐induced response.

The Pre‐S1 region has also attracted attention, and Zheng et al.^67^ have analyzed **W682**, a residue that is conserved within several TRP families. The mutant **W682A** TRPM8 showed a reduced channel opening probability, and a similar behavior was observed with **R998A**, a residue at the TRP domain close in space to **W682**. Moreover, either of these mutants dismissed the Ca^2+^ entry in response to menthol. A role of **W682** and **R998** in the mediating the interaction between TRPM8 N‐ and C‐termini was suggested. Besides, as R998 is within the PIP2 binding pocket, it was proposed that PIP2 stabilizes the interaction between both termini.

### 2.2 Mutations in the VSLD region

Bandell et al.^68^ studied mouse TRPM8 (*mm*TRPM8) and generated a random mutant library through error‐prone polymerase chain reactions. Subsequent ratiometric Ca^2+^ imaging and whole‐cell patch‐clamp experiments led to the identification of several mutations that, while maintaining a functional channel, altered the response to menthol. Among them, Y745, located in S1, was shown to be a crucial residue in modulating responses to the agonist. Of note, in this and other publications, Y745 is indicated as being a residue of S2, but the resolution of TRPM8 cryo‐EM structures showed that it is located at S1.^13^, ^57^, ^58^ The replacement of this Tyr for His (**Y745H**) abolishes the response of the channel to both menthol and icilin, however, does not affect responses to either cold or voltage dependence of the channels, or PIP_2_ sensitivity, compared to wild‐type (WT) *mm*TRPM8. Additionally, these ligands could not potentiate cold responses on the **Y745H**‐*mm*TRPM8, even when applied at high concentrations.^68^ Interestingly, **Y745A** *mm*TRPM8 showed no sensitivity to menthol, whereas the substitution of Tyr by Phe (**Y745F**), although affecting menthol sensitivity at a low concentration, showed menthol responses at high concentrations, suggesting that not only the hydroxyl group but also the presence of an aromatic ring is needed for menthol activity. Although this behavior is suggestive of direct interaction of Y745 with the compounds, other possibilities could explain these results, as the inability of **Y745H** mutant to transduce conformational changes upon ligand binding. Following studies replicated these results and advanced the knowledge of Y745 substitutions. The group of F. Viana reported that **Y745H** mutant channel was insensitive to menthol, and that this mutation does not affect cold‐evoked response or voltage dependency.^70^ As well, they were able to identify a slight reduction of apparent gating charge and no menthol‐induced decrease in gating charge for the mutant, contrary to that observed in WT‐*mm*TRPM8.

Beccari et al.^69^ performed single point mutations and generated the **Y745A** variant. The analysis of the effect of this mutant on icilin and the menthol analog Cooling Agent 10 (Supporting Information: Figure S1) showed that this mutation renders a channel insensitive to these compounds. A similar behavior was observed for the **I746A** mutant, although a residual activation was still detected.

Y745 was found to be an important residue for other exogenous ligands, as the immunosuppressant macrolide tacrolimus, a TRPM8 agonist able to potentiate the cold‐induced responses in human TRPM8 (*hs*TRPM8).^74^ **Y745H**‐*mm*TRPM8 exhibited tacrolimus‐induced activation of the mutant channel similar to the WT, but no potentiation of cold‐evoked responses was reported. The anthelmintic drug Praziquantel is a TRPM8 partial agonist that showed a behavior similar to menthol in the **Y745H**‐*hs*TRPM8 variant, where both were unable to elicit a response.^71^ Several TRPM8 antagonists have been analyzed by Viana and colleagues, namely BCTC, SKF96365, capsazepine, clotrimazole, and econazole, on the **Y745H**‐*mm*TRPM8 mutant.^70^ BCTC was capable of blocking channel responses to cold and voltage as it did in the WT‐*mm*TRPM8, an effect exerted through a reduction in maximum conductance and elevation of half‐activation voltages to more positive potentials. SKF96365, on the contrary, failed to inhibit cold‐evoked responses, even at high concentrations. Capsazepine, clotrimazole, and econazole retained activity in the **Y745H**‐*mm*TRPM8 mutant, although significant reductions of their potencies were detected. Authors hypothesized that this might be due to Y745 directly interacting with menthol and SKF96365, with docking studies supporting the hypothesis of Y745 as a key residue of their binding pockets.

The above studies reported the **Y745H** variant as insensitive to menthol. However, the determination of menthol binding through indirect measurements opened the possibility that the substitution could affect either menthol interaction or downstream gating steps. The observation by Voets et al.^73^ of no [^3^H]menthol displacement when incubated the **Y745H** mutant channel in the presence of cold menthol, in contrast with WT‐TRPM8, was suggestive of Y745 being involved in the direct binding. On the other hand, Van Horn and colleagues determined the direct menthol binding to the isolated VSLD (S1–S4 segment), through different biophysical measurements, including Nuclear Magnetic Resonance, far‐ultraviolet, circular dichroism, and microscale thermophoresis.^72^ They reported binding of menthol on both WT‐VSLD‐*hs*TRPM8 and **Y745H**‐VSLD‐*hs*TRPM8, with no significant differences in binding affinity, as well as minor, insignificant structural differences among WT and **Y745H** S1‐S4 segments. On the light of these results, Y745 was suggested to play a role in the coupling of menthol binding and channel gating, via conformational changes upon ligand‐binding.

**Figure 4:**
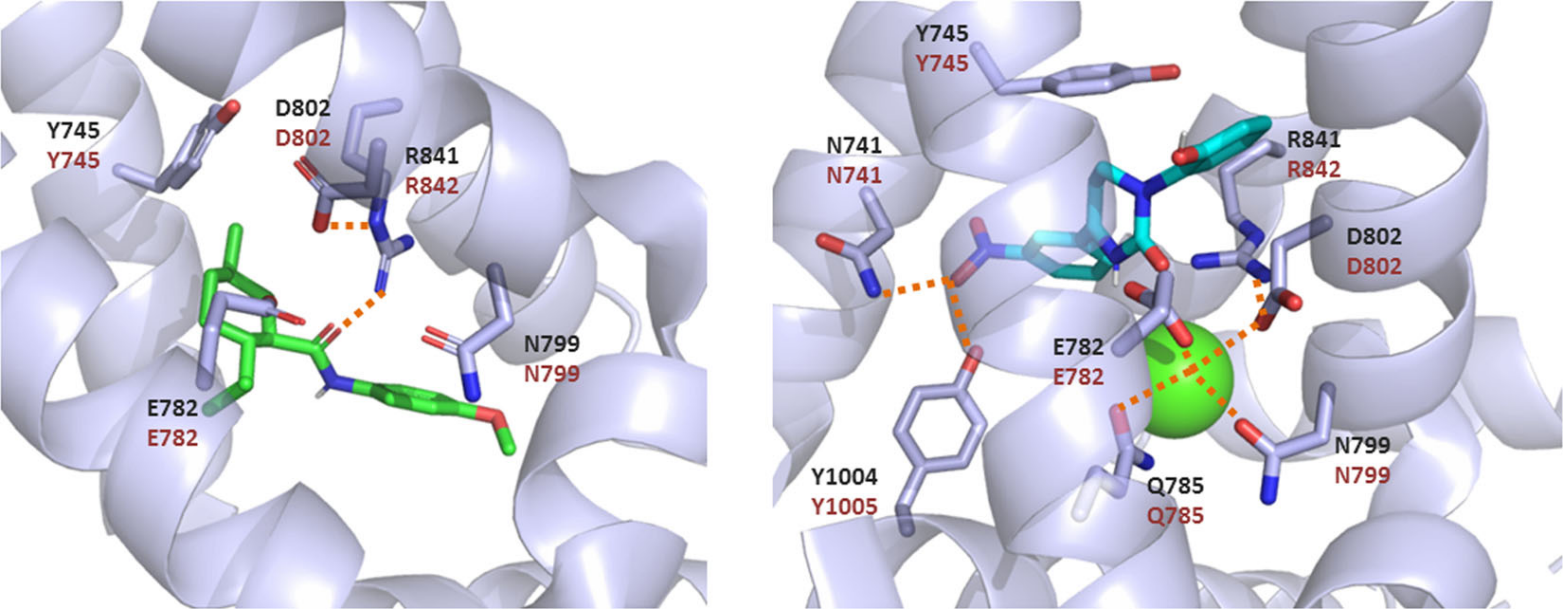
View of *fa*TRPM8 in complex with ligands. On the left, view of *fa*TRPM8 in complex with menthol analog WS‐12 (PDB ID 6NR2), on the right, view of *fa*TRPM8 in complex with icilin (PDB ID 6NR3). For clarity only residues included in the text are shown. Polar interactions are depicted as orange dashes lines. Ca^2+^ ion is depicted as a green sphere. Residue numbers in *fa*TRPM8 (dark gray), in *hs*TRPM8 (brown) [Color figure can be viewed at wileyonlinelibrary.com]

In 2019, the group of S.‐Y. Lee determined the cryo‐electron microscopy structures of an avian TRPM8 channel in complex with menthol analog WS‐12 and with icilin (PDB IDs 6NR2 and 6NR3, respectively).^13^ As previously suggested by mutagenesis studies, the binding pocket is located within the VSLD and the TRP domain. In these structures, the Y745 residue in S1 (Y736 in the *pm*TRPM8 sequence) is within the binding pocket of these two cooling agents (Figure 4). It is worth to point out that Y745 is close to the menthol‐like moiety of WS‐12. Regarding the complex with icilin, besides PIP_2_, a Ca^2+^ ion is present in this structure, which shed light on the requirement of intracellular Ca^2+^ ions for TRPM8 activation by icilin, but not by menthol or cold.^1^

A well‐characterized segment is the one spanning between W798 and F815, which corresponds to VSLD helix S3, in which crucial residues for ligand binding have been identified. Within this segment there is a residue that confers sensibility to icilin in mammalian species, G805, but is naturally absent in the avian channel.^77^ Indeed, the group of D. Julius studied **G805A** mutant in the rat TRPM8 (*rn*TRPM8), showing that this residue is necessary for icilin sensitivity and icilin‐induced cold potentiation, without affecting menthol and cold responses. The importance of this residue is in agreement with studies by Kühn et al.^89^ that substituted the **803‐TLGLFY‐808** sequence of *hs*TRPM8 by the corresponding sequence of its closest human homolog TRPM2 channel, **VGAILL**, a channel insensitive to icilin, or to any other TRPM8 activators. This mutant, as expected, showed no responses to icilin, however, menthol activation and menthol‐induced cold potentiation, as well as responses to menthol analogs (WS‐12, CPS‐154, CPS‐369), temperature and voltage were similar to WT‐*hs*TRPM8. Furthermore, icilin, when applied prior (or shortly after) an activating stimulus (cold, menthol, WS‐12, or CPS‐369) was capable of preventing the response in the mutant channel. Additionally, and unlike icilin‐evoked activation, this effect was completely Ca^2+^‐independent. Finally, the effect of the partial agonist *Praziquantel* was also studied in **G805A**‐*hs*TRPM8, and different to icilin, it exhibited WT‐like responses.^71^ Once established the importance of G805 in icilin sensitization, Julius and colleagues performed alanine scan mutagenesis around this residue, to identify further residues that might be also involved.^77^ Their results showed the importance of N799 and D802, as mutations to Ala led to icilin insensitive variants, while being sensitive to cold or menthol. However, two different results were observed on cold responses potentiation by icilin in the absence of extracellular Ca^2+^: while **D802A**, similarly to the G805A variant, abrogated icilin‐induced potentiation, **N799A** showed WT‐like responses. The authors suggested that the **N799A** variant might be more important for Ca^2+^ interaction, whereas the others, for icilin binding. Further studies analyzed the substitutions of D802 by other amino acids (Tyr, Ser, Glu, Asn, Gln, His, and Lys), reporting insensitivity of the mutant channel to icilin, with WT‐like responses to cold and menthol, thus showing the importance of Asp residue in this position for TRPM8 icilin sensitivity. The motif N‐x‐x‐D (corresponding to residues 799 to 802 in the *hs*TRPM8) appears highly conserved in the family of voltage‐dependent cation channels, as well as other voltage‐independent ones.^78^ Thus, Winking et al. further explore the role of the N‐x‐x‐D motif analyzing the effects of swapping N and D residues on responses to cold, menthol and icilin, preparing *hs*TRPM8 **N799D**, **D802N** variants and the double mutant **N799D** + **D802N**. There was no response to icilin in neither of the mutant channels. However, they differed in the response to cold and menthol. In both electrophysiology and Ca^2+^ imaging experiments, menthol‐evoked responses were significantly impaired in the **N799D**‐*hs*TRPM8 channel, whereas **N799D** + **D802N** and **D802N** showed increased currents. Subsequent Western blots analysis of the **N799D** mutant showed no band for the matured *N*‐glycosylated channel, which is suggestive of biogenesis impairment. On the other hand, responses to cold were reduced in all mutants. These data indicated that N799 and D802 residues are relevant to icilin, menthol and cold sensitivity. Furthermore, the D‐X‐X‐N central residues were analyzed by substituting those in the *hs*TRPM8 (NVMD) for those present in the human TRPM2 (NKLD), resulting in the double mutant **V800K** + **M801L**. This mutant exhibited a marked decrease in sensitivity to menthol and cold, but also absence of the matured N‐glycosylated isoform, as for **N799D**, which might be attributed to defects in channel assembly or plasma membrane expression.

Beccari et al. reported the discovery of a novel naphthyl antagonist (Supporting Information: Figure S1) capable of preventing the in vitro activation of *hs*TRPM8. They explored the effects of this antagonist and agonists icilin and cooling agent 10 on **N799A** and **D802A** *hs*TRPM8 mutants.^69^ Their results showed that both mutations completely abolished *hs*TRPM8 activation by icilin, replicating prior results. Cooling agent 10, however, was not affected by the **N799A** mutation, while the **D802A**‐*hs*TRPM8 variant showed reduced potency. When the inhibition by the naphthyl antagonist of the cooling agent 10‐induced activation was tested, potency in **D802A** channel was reduced compared to WT, while in the **N799A** mutant, it showed enhanced antagonist potency. It was also observed that, in the **I746A** variant, the naphthyl antagonist caused a decreased inhibition on the icilin response. Docking studies showed that only D802 is participating in a hydrogen bond with the naphthyl antagonist. As in the resting state N799 might be involved in a hydrogen bond with D802, the replacement of N799 by Ala prevents this hydrogen bond, suggesting that in the **N799A** mutant, residue D802 may be more prone to interact with the antagonist. These studies are indicative of the naphthyl derivative binding into the ligand binding pocket of TRPM8. The effect of **N799A** mutant channel has also been studied regarding the macrocycle tacrolimus, which showed a similar behavior as in WT‐*mm*TRPM8, where this agonist activated the channel and potentiated cold‐evoked responses.^74^

The structure revealed by cryo‐electron microscopy of *fa*TRPM8 (PDB ID 6NR3) shed light regarding the role of N799 and D802 residues. It is worth to point out that the avian channel is insensitive to icilin, thus, to induce sensitivity, a mutation of A805 (S3) to Gly is necessary. Neither N799 nor D802 residues are involved in a direct interaction with icilin, rather, they are participating in Ca^2+^ coordination, together with E782 and Q785 (Figure 4).^13^ As already mentioned, Ca^2+^ is necessary for sensitivity to icilin.^1^ The group of S.‐Y. Lee, on the base of their TRPM8 3D structures, proposed that upon Ca^2+^ binding there is a conformational change that enlarges the VSLD to allow the interaction with icilin.^13^ On the other hand, it is interesting to indicate that these residues are not involved in binding to menthol analog WS‐12 (PDB ID 6NR2). Subsequent studies by the group of Julius analyzed whether Ca^2+^ binding might be the reason for desensitization of menthol elicited response, and showed that **Q776A** or **N790A** *pm*TRMP8 (corresponding to Q785 and N799 in *hs*TRPM8) diminished desensitization.^58^

Further insights into residues involved in icilin sensitivity were provided by **H844A** mutant. H844 is a positive residue at S4, which is within the binding pocket of icilin, but not WS‐12. As expected, the **H844A** mutant strongly abolishes activation of the channel by icilin, on the contrary, no effect is observed for WS‐12 activation. H844 has been proposed to be behind the difference in pH sensitivity between these two agonists, with icilin being affected by intracellular pH.^13^

The unnatural fluorescent amino acid ANAP has also provided insights into menthol binding. Xu et al.^75^ incorporated this residue in 73 sites through the VSLD. The emission of the ANAP residue shifts to higher wavelengths in hydrophilic environments. They found 39 sites which tolerated the substitution (maintaining menthol‐elicited currents), nine of those being involved in conformational changes upon menthol binding. Imaging experiments with ANAP showed a redshift of ANAP emission peak for **L774ANAP**, **V791ANAP**, and **S824ANAP** variants, which map the S1–S4 segment. Besides, as it is indicated below, six residues within the pore region also showed a shift of the emission peak. These results, together with docking studies, provide insights into menthol binding pocket, and are indicative of conformational rearrangements within the TM domains induced by menthol.

Kühn et al.^76^ focused in the analysis of the effect of substitutions of D802, in conjunction with mutation of R842, to elucidate the interplay between S3 and S4 and its role in *hs*TRPM8 gating. A previous model by Pedretti et al.^95^ had already postulated the crucial role of both residues in channel gating: the binding of an agonist would lead to a conformational change in S3, which would position D802 in close proximity to R842 (S4), allowing their electrostatic interaction and the subsequent movement of S4. This would finally lead to conformational changes in the PD that would allow cations passage. To experimentally test this hypothesis, several *hs*TRPM8 variants were prepared. First, the effect of charge reversal substitutions in R842 was studied, with **R842E** and **R842D** mutants, which failed to respond not only to icilin, but also to menthol, voltage, and cold. However, they both lacked the mature *N*‐glycosylated channel, that might prevent correct integration into the membrane, which may be responsible for the absence of activation. To test the hypothesis of the electrostatic interaction between residues D802 and R842, the activity of the double charge reversal mutants **D802R** + **R842E** and **D802R** + **R842D** was also analyzed. Both variants recovered the glycosylated isoform and displayed a voltage‐dependency profile as the WT‐*hs*TRPM8. Moreover, whole‐cell patch‐clamp and Ca^2+^‐imaging experiments indicated a partial recovery of the responses to cold and menthol. Nonetheless, for menthol, the recovered response was significantly greater in the case of the **D802R** + **R842D** mutant. However, both failed to respond to icilin. The existence of a salt bridge between D802 and R842 has been subsequently corroborated by TRPM8 cryo‐EM structures (Figure 4).^13^, ^57^, ^58^ Moreover, when studying the single mutant **D802R**, which in accordance with the proposed hypothesis should display a similar phenotype as **R842D/E**, it was observed that, as expected, the mutant did not respond to icilin.^76^ However, channel glycosylation, voltage‐dependency, cold, and menthol responses were almost intact. It was suggested that in mutant **D802R** this residue may be interacting with a negatively charged residue in S4 and, thus, the activity in the **D802R** + **D835R** mutant was analyzed, which was fully nonfunctional. As above indicated, subsequent cryo‐EM studies explain this behavior based on the involvement of D802 in Ca^2+^ coordination.^13^ On the other hand, the single mutant **D835R** lacked the mature *N*‐glycosylated isoform, that might led to assembly defects, and thus to a nonfunctional channel. Additional charged residues were analyzed, as double mutants **D796R** + **R842D** and **D802N** + **R842D**. **D796R** + **R842D** showed no responses to cold, menthol and icilin, no voltage‐dependent activation, and absence of the mature glycosylated form. The single mutant **D796R** maintained cold and menthol sensitivity, and the fully glycosylated isoform, but did not retain icilin‐evoked responses; nevertheless, icilin was able to abrogate the response to menthol. The cryo‐EM structure of *pm*TRPM8 (PDB ID 6O77) shed some light into these observations, as this residue, D787 in *Parus mayor*, is participating in a hydrogen bond with the backbone amino group of N790 (N799 in *h*TRPM8), a residue involved in Ca^2+^ coordination, which as mentioned is essential for icilin sensitivity.^58^ On the other hand, the **D802N** variant showed, again, channel glycosylation, voltage‐dependency, and menthol‐ and cold‐evoked responses, lacking icilin sensitivity, while the **D802N** + **R842D** mutant only slightly recovered the detrimental effect of the single **R842D** substitution, displaying partial voltage sensitivity, and small menthol‐induced currents. Finally, positive charged residues in the S4 were analyzed by mutation to the corresponding ones in the Shaker channel, **F839R** + **H845R** and **F839R** + **H845R** + **T848K**. Although expecting enhanced voltage sensitivity, as in Shaker K^+^‐channels, both variants provoked a decrease in voltage current amplitude and required higher potentials to elicit responses. Additionally, Ca^2+^ imaging experiments showed no differences on menthol‐evoked responses, but these variants could not respond to icilin. These studies led the authors to suggest that there is cooperation between S3 and S4 segments for TRPM8 gating.

Selescu et al.^79^ analyzed the effects of camphor, a molecule able to potentiate cold sensation, on TRPM8 activity, and compared it with menthol, eucalyptol, and icilin. As TRPM8 orthologs show different sensitivity to agonists,^9^, ^77^, ^80^, ^96^ the effects on *rn*TRPM8, *hs*TRPM8 and chicken TRPM8 (*gg*TRPM8) were studied. Rat and human TRPM8 were activated by camphor, but no response was shown on the icilin‐insensitive *gg*TRPM8. Due to the shared insensibility of the avian TRPM8 to icilin and camphor, the effect of camphor activity on the *hs*TRPM8 **D802A** mutant, which has been reported to turn human channel insensitive to icilin,^77^ was analyzed. Interestingly, **D802A**‐*hs*TRPM8 was activated by camphor as in the WT channel. They further determined that coadministration of either camphor or eucalyptol and menthol resulted in a significant reduction in menthol‐induced currents in the human, rat, and avian TRPM8 channels. This behavior is suggestive of a bimodal action of camphor in TRPM8, showing activation in the mammalian channels and inhibitory activity on menthol responses.

Voets et al.^73^ analyzed positively charged amino acids within S4 and the S4–S5 linker, Lys and Arg, together with H845, as His may be protonated depending upon the environment. To study residues that might contribute to gating charge, neutralizing and charge‐conserving substitutions were considered.^97^ In voltage‐gated ion channels, gating charge allows the movement of the voltage sensor S4 helix.^98^ As **R851A** yielded a nonfunctional channel, the final studied mutations were **R842A**, **R842H**, **R842K**, **H845A**, **R851Q**, **R851K**, **K856A**, **K856R**, **R862A**, and **R862K**.^73^ Among several parameters, voltage, temperature, and menthol binding were studied for these mutants. Of note, the subsequent cryo‐EM structures of TRPM8 showed that residues K856 and R862 (which correspond to K855 and R861 in *fa*TRPM8, and K846 and R852 in *pm*TPRM8) are at the bottom of S5, and thus take part of the pore region.^13^, ^57^, ^58^ Concerning voltage‐dependence, **R842K**, **R851K**, and **R862K** mutants, which conserve the charge, maintained WT‐like half activation voltages, while for **R842A**, **K856R**, and **R862A** the voltage dependence was shifted to exceptionally positive voltages, and only **K856A** was shifted toward more negative voltages. Additionally, they estimated gating charge on the mutants, and reported reductions on **R842A** and **K856A** mutant channels, which may account for most, but not total, of the gating charge of TRPM8. Accordingly, charge‐conserving substitutions in these positions (**R842K** and **K856R**) had no effect on the gating charge when compared to the WT channel. Regarding the temperature threshold for activation, only **K856A** variant moved toward warmer temperatures, while **R842A**, **H845A**, **R851Q**, **R862A** *hs*TRPM8 mutants exhibited lower (cooler) activation thresholds than the WT‐*hs*TRPM8. These results, taken together, support the notion that voltage and temperature dependence are tightly linked. Finally, similar menthol sensitivity was found for **H845A**, **R851Q**, **R862A**, and **K856R** mutants compared to WT‐*hs*TRPM8. On the contrary, lower sensitivity was observed for the substitution of **R842** by Ala, Lys or His, whereas **K856A** variant showed higher sensitivity. Subsequent studies with the two variants that affect menthol sensitivity showed that they also influence its affinity. Thus, radioligand studies with tritium‐labeled menthol showed that in **K856A** and **R842A** mutants, menthol exhibited lower and higher IC_50_ values, respectively, compared to WT TRPM8. In a subsequent study by Rath et al.,^72^ based on microscale thermophoresis and NMR, it was postulated that R842 is not involved in direct menthol binding, and that the reduced affinity could be explained by the residue playing a role in coupling agonist binding to pore gating. The contradictory results might be due to differences in the performed experiments, and to the fact that in the latter study the binding of menthol was measured at the *hs*TRPM8 S1–S4 construct (sensing domain), whereas Voets et al.^73^ used cells transfected with the full‐length *hs*TRPM8.

The group of D. Julius resolved several cryo‐EM TRPM8 structures and modeled the transition of the closed antagonist‐bound state to the Ca^2+^‐bound desensitized state.^58^ These authors indicated that R832 *pm*TRPM8 (corresponding to R842 in *hs*TRPM8) is participating in the transition of an α‐helix turn in S4 to a 3_10_ helix by interacting with Y736 and D793 (Y745 and D802 in the human channel), which led to relevant conformational changes.^58^ On the other hand, as mentioned previously, the group of S.‐Y. Lee^13^ resolved the cryo‐EM structure of *fa*TRPM8 bound to icilin (PDB ID 6NR3) and the menthol analog WS‐12 (PDB ID 6NR2). An interaction between the sidechain of R841 (R842 in *hs*TRPM8) and the carbonyl oxygen of icilin or WS‐12 was observed in both structures (Figure 4).^13^ In the *fa*TRPM8 apo form (PDB ID 6BPQ), residue R841 (R842 in *hs*TRPM8) is forming hydrogen bonds with D802 and E782, while the latter is not present in the structure in complex with WS‐12. This seems to indicate that WS‐12 disrupts the packing of these three residues, which might lead to a conformational rearrangement of the TRPM8 channel. Recently, another model of menthol binding to TRPM8 is also suggestive of the presence of a hydrogen bond between its hydroxyl group and R842.^75^ This latter structure also shed light regarding the involvement of R850 of *fa*TRPM8 (R851 in *hs*TRPM8) in the interaction with PIP_2_. Differently to the ligand free or WS‐12‐TRPM8 structures, in the complex with icilin (PDB ID 6NR3) there is a rearrangement that positions R850 close to PIP_2_ phosphate groups, thus allowing their interaction. Residues from other domains are also implicated in the interaction with PIP_2_, as *fa*TRPM8 K605, R688 and R997 (this last residue corresponding to *hs*TRPM8 R998).

Although Voets et al.^73^ have shown that some basic residues are involved in menthol activation, as R842 or K856, these residues are conserved in heat activated channels, as TRPM2, TRPM4, and TRPM5, which are insensitive to menthol. To investigate further residues implicated in menthol sensitivity, three chimeric channels were constructed by substituting small segments of the *hs*TRPM8 S4 by those of TRPM5. Only one of them, where the segment **848‐TVSRN‐852** was replaced by **AIHKQ**, was functional. This chimeric channel is activated by cold, with properties similar to WT TRPM8, whereas it showed lower sensitivity to menthol with higher EC_50_ values, and a decrease in [^3^H]menthol binding.

There are several other residues within the VSLD that are also involved in cold‐evoked responses, but they will be discussed below, together with those of the pore regions that are also important in temperature sensing.

### 2.3 Pore region mutations

The PD includes helices S5 and S6 along with the small pore helix. It is worth to point out that mutations within this region are able to switch TRPM8 selectivity from cations to anions. Kühn et al.^99^ showed that replacement of V976 by Lys lead to an anion channel that displays larger currents at room temperature compared with WT, and showed no desensitization. They suggested that the lack of desensitization in these channels, which do not allow Ca^2+^ entry, might support the hypothesis that this process is regulated by Ca^2+^ influx. Furthermore, **V976D** mutant channel is unable to conduct current upon cold, menthol, or icilin stimulation. In the cryo‐EM structure of PDB ID 6O77, a desensitized state of the channel, this Val (V970 in the avian receptor) reduces the radius of the lower gate.^58^ V976 variants have also been of utility to study the involvement of TRPM8 channels in a metabotropic Ca^2+^ signaling pathway. Klasen et al.^86^ hypothesize that this metabotropic pathway is independent from Ca^2+^ influx through the TRPM8 channel, among others evidence, they analyzed the chloride‐conducting **V976K** and the nonconducting **V976D** TRPM8 variants, that were able to show metabotropic Ca^2+^ signaling. This pathway, that was independent of the ionotropic signaling pathway, may implicate activation of a heterotrimeric G protein of the G_q_ family by TRPM8.

The PD has also crucial residues involved in TRPM8 biogenesis and function. In 2006, two groups analyzed possible glycosylation sites and identified N934 residue, at the extracellular loop between S5 and S6, as a glycosylation site, since **N934Q** mutant showed a single Western‐blot band corresponding to the nonglycosylated isoform.^83^, ^84^ Dragoni et al.^83^ measured intracellular calcium levels for **N934Q** mutant and reported a similar response to menthol or icilin as for the WT channel, as well as no modification of the threshold of activation for cold stimulus, though the amplitude of the response to cold was smaller. Erler et al.^84^ analyzed the influence of **N934Q** mutant on menthol‐induce currents, and although the shape of the current‐voltage relationship was similar to WT, there was a smaller response to menthol. In both studies biotinylation assays were performed, and indicated a lower level of **N934Q** variant on the surface in comparison with WT. Nevertheless, Erler et al. suggested that glycosylation is not required for trafficking to the plasma membrane. In 2012, the group of F. Viana also studied the **N934Q** mutant by Ca^2+^ imaging and electrophysiological experiments. They also analyzed *N*‐glycosylation in native cold thermoreceptor neurons, a more physiological context, rather than using heterologous expression systems.^20^ In agreement with previous studies, a reduction in the response to cold and menthol was found but, unlike them, similar surface expression levels as those of WT were observed by biotinylation experiments. Thus, the observed reduction in response seems due to lower sensitivity to cold or menthol of the mutant channel. They analyzed whether the loss of the negative charge of the *N*‐glycosylation in the **N934Q** mutant might be behind its different behavior, and in accordance with this hypothesis, **N934K** TRPM8 showed similar responses as those of the **N934Q** mutant, while **N934D** TRPM8 partially recovered the response to cold and menthol.

**Figure 5:**
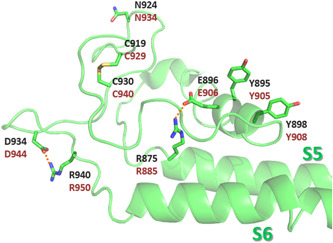
View of the pore domain residues that have been mutated (*pm*TRPM8 structure, PDB ID 6O77). Residue numbers corresponding to *pm*TRPM8 (dark gray) and to *hs*TRPM8 (brown). Polar interactions are depicted as dotted orange lines [Color figure can be viewed at wileyonlinelibrary.com]

Close to this glycosylation site, two Cys residues, C929 and C940, have shown to be crucial for *mm*TRPM8 assembly and activity. Upon mutation of any of them to Ala, or both (**C929A** + **C940A**), the channel was unable to respond to icilin or menthol stimulation.^83^ The lack of activity cannot be attributed to faulty translocation to the plasma membrane, as these mutants can be biotinylated at the cell‐surface. Similar results were obtained by Bidaux et al.^81^ with **C940G** or **C940R** TRPM8 mutant channels. Dragoni et al.^83^ hypothesized that these residues could be involved in intramolecular disulfide bond formation and subsequent generation of a protein loop with a carbohydrate linked to N934. When analyzing the cryo‐EM structures of TRPM8, the region comprising these Cys, in general, has not been resolved, with the exception of the pmTRPM8 structure of PDB ID 6O77 (calcium‐bound desensitized state). In this structure, the proposed disulfide bond is observed (Figure 5, corresponding to C919 and C930 in the *P. major* channel PDB ID 6O77).^58^

Besides C940, Bidaux et al. analyzed other residues within the S5‐linker‐S6 region, using molecular modeling and mutagenesis studies, particularly those within the selectivity filter (SF) ^917^SDVD^920^.^81^ The Asp residues within SF are conserved in the TRP family,^100^, ^101^, ^102^ although D918 might be replaced by Glu, and Asp residues from each subunit form two rings known as the DDDD rings. Replacement of these Asp residues by Glu did not affect TRPM8 activity, neither the substitution of D918 by Ala or Asn. However, **D920A** or **D920N** mutant channels experienced a drastic decrease in TRPM8 current in response to cold, icilin and menthol. All these mutants were detected at cell surface, and did not show modified electrophysiological properties or ion selectivity. For double mutants **D918N** + **D920N** and **D918A** + **D920A**, current was only detected in the latter in response to cold, menthol, and icilin, albeit with a large decrease compared to WT. The authors suggested that the DDDD rings are collaborating in the stabilization of the pore and, as such, Asp mutations likely led to destabilization of the ion conduction. They also analyzed **V919I** mutant, which did not affect TRPM8 currents in response to cold, menthol, or icilin. Next, the P‐helix between S5 and S6 was studied, in particular, residue Y905, which according to their model stabilizes this helix through stacking interactions with Y908. In agreement with this hypothesis, the Y905F mutant led to an active channel, whereas **Y905A** prevented activation in response to cold, menthol or icilin. Interestingly, the replacement of Y908 by Ala or Trp led to a decreased response to cold and menthol, but did not affect icilin activity. However, **Y908F** mutant showed a similar behavior as WT. Moreover, coexpression of **Y908A** TRPM8 mutant with WT channels decrease sensitivity to cold, but not to menthol, suggesting that this helix is involved in responses to cold and menthol but not to icilin. E906, also in the P‐helix, forms a salt bridge with R950, at the S5‐S6 loop, in the indicated model. **E906A** and **R950E** mutants are inactive, whereas partial activity is observed for **E906Q** channel. In the cryo‐EM structure of PDB ID 6O77, similarly to this model, Y905 and Y908 are in two consecutives turns of the pore helix (Figure 5, corresponding to Y895 and Y898 in *pm*TRPM8, respectively).^58^ On the other hand, as shown in this structure of pmTRPM8, in the desensitized state, residues E896 and R940 (E906 and R950 in *hs*TRPM8), are not involved in a salt bridge between them. However, their sidechains are implicated in salt bridges with other residues in the channel, namely, the side‐chains of R875 and D934 (R885 and D944 in *hs*TRPM8), respectively (Figure 5).

The relevance of PD for cold sensitivity has also being observed in the comparison between mouse and chicken TRPM8 channels by the group of R. Madrid.^80^ *gg*TRPM8 channel display higher sensitivity to menthol than *mm*TRPM8. On the contrary, cold‐induced response is larger for the *mm*TRPM8. Through the use of these orthologues to build chimeras, and Ca^2+^ imaging and patch‐clamp experiments, these authors suggested that for their chimeras the PD is important for cold sensitivity but do not affect menthol responses. By analyzing sequence differences between these orthologues, they mutated **R897E** in *mm*TRPM8 (E887 in *gg*TRPM8, E888 in our alignment Supporting Information: Figure S2, sequence retrieved from UniProt database). **R897E** *mm*TRPM8 channel showed a similar response to menthol, but a decrease of cold‐evoked currents compared to WT *mm*TRPM8. Further regions had been identified in the study of Matos‐Cruz et al.,^87^ that aims to get insights into the molecular mechanism involved in cold tolerance during hibernation, through the analysis of TRPM8 channels from 13‐lined ground squirrels and Syrian hamsters.^87^ The comparison of TRPM8 expression in neurons of these two hibernators with mouse suggested that cold tolerance cannot be attributed to a decrease in TRPM8‐positive neurons. The presence of functional channels was demonstrated by the fact that squirrel neurons were sensitive to icilin, similarly to those of mouse, however, squirrels showed a lower response to cold. Moreover, the sensitivity to icilin and menthol of squirrel and hamster was also similar to rat, as shown by EC_50_ values, but squirrels and hamster showed virtually no response to cold below 20°C. The transmembrane core region seems to be responsible for cold sensitivity, as demonstrated through chimeras and mutagenesis studies. Six residues in squirrel TRPM8 (*it*TRPM8) were identified that, when replaced by the corresponding rat ones, did not affect icilin response, but led to sensitivity to cold: **H726Y**, **A762S**, **P819S**, **A927S**, **H946Y**, and **S947N**. These residues are spread over the TRPM8 core, the first three within the loops connecting transmembrane regions, and the last three located at the pore region.

A recent work by Yang et al.^82^ also focuses on the importance of the PD for cold sensing. Studying two species that live in very different environments, the African elephant (*Loxodonta africana*) and the emperor penguin (*Aptenodytes forsteri*), it was observed that the latter had a small cold activated TRPM8 current at 6°C, whereas the former showed a strong current. As large variations in enthalpy (ΔH) and entropy (ΔS) are associated with TRPM8 cold activation, they were quantified for both species (measuring temperature–current relationship), and found that the variation was greater for the African elephant, while both channels showed similar voltage dependence. By analyzing nonconserved pore residues and mutagenesis studies, these authors identified residue 919 as a key one. Thus, **V919Y** elephant TRPM8 channel (*la*TRPM8) led to a decrease in the cold‐evoked current, and a decrease in ΔH and ΔS. On the contrary, the reverse mutation **Y919V** in penguin TRPM8 (*af*TRPM8, Y914 in our alignment Supporting Information: Figure S2, sequence retrieve from UniProt database) led to an increase in the current, ΔH and ΔS. Based on the hypothesis that a transit of a hydrophobic side‐chain to a more water‐exposed medium might lead to an increase in cold response, ANAP imaging experiments were performed. These studies allowed the identification of three mutants, **G925ANAP**, **L943ANAP**, and **L947ANAP** (elephant TRPM8 numbering, which correspond to *hs*TRPM8 residues G921, L939, and L943) which, upon cold activation, showed a redshift in ANAP emission, which might be indicative of their higher exposure. In addition, the incorporation of higher hydrophobic amino acids, like ANAP or Ile, led to an increase in ΔH and ΔS, and higher cold sensitivity, whereas more hydrophilic residues, such as Gln or Lys, led to a decrease in ΔH and ΔS and in cold sensitivity. In summary, these studies suggest that side‐chain hydrophobicity and solvent accessibility of certain TRPM8 residues are involved in adaptation to temperature.

Interestingly, the PD has also been implicated in the conformational rearrangement induced by menthol binding to TRPM8, together with other transmembrane regions. Xu et al.^75^ analyzed these conformational changes by substituting residues with the fluorescent amino acid ANAP. Upon menthol binding, they found five mutants in PD that induce a redshift in ANAP emission, and another one a blueshift, reflecting a change in the environment of the ANAP residue. The authors suggested conformational changes around the following six residues: A875, D920, L939, P958, and S966, which showed a redshift, and T922, displaying a blueshift in ANAP emission. Regarding S966, the comparison of the closed and desensitized TRPM8 structures in PDB IDs 6O77 and 6O6R showed that this residue (S956 in pmTRPM8) is within a segment that undergoes a conformational change from α‐ to π‐helix.^58^

### 2.4 C‐terminal domain

The role of the whole *C*‐terminal domain has been analyzed by the group of R. Latorre by using chimeras, in which this domain is switched between TRPV1, the heat receptor, and TRPM8, the cold receptor.^90^ These chimeras showed that the *C*‐terminal domain is modular and confers temperature sensitivity. Thus, the chimera with TRPM8 *C*‐terminus is activated by cold, and contrarily, that with the TRPV1 *C*‐terminus is activated by heat. Regarding regulation by PIP2, the chimera with the TRPV1 *C*‐terminus and TRPM8 core showed an inhibitory response, whereas for wtTRPM8 there was a positive modulation. Furthermore, the replacement of TRPM8 *C*‐terminus by that of TRPV1 does not affect menthol sensitivity, and the channel is insensitive to capsaicin, the TRPV1 ligand. More recently, this group carried out a progressive deletion of the *C*‐terminal domain, and found that the last 36 residues deletion led to a channel with reduced temperature sensitivity.^91^ These studies suggested that the *C*‐terminal domain folds in response to cold, and that TRPM8 gating is coupled to the folding‐unfolding of this domain.

The *C*‐terminal domain also has a role in tetramerization through the coiled‐coil region, which is necessary for channel assembly. Tsuruda et al.^103^ expressed a TRPM8 mutant lacking residues 1064–1104 (that were predicted as having a high probability of coiled‐coil structure), and subsequent patch‐clamp and Ca^2+^ imaging studies showed no response to cold or menthol in this deletion mutant. This lack of functionality might be attributed to the lack of expression of this mutant, as shown by Western blot analysis experiments. Within the coiled‐coil segment Erler et al.^84^ analyzed the **L1089P** mutant, that might alter the helical structure of this domain. **L1089P**‐TRPM8 channel showed a great reduction in oligomerization, reduced surface expression, and fails to form a functional channel. Subsequent studies by C. Phelps and R. Gaudet showed that the deletion of the *C*‐terminus in TPRM8‐∆C1070 led to a mutant channel able to tetramerize and localized in the membrane. These authors indicated that their insect cells overexpression system might help to form tetramers even without the C‐terminal coiled‐coil.^60^ Nevertheless, this deletion mutant prevented proper gating of the channel, indicating that this region is also necessary for channel activation.

The C‐terminus contains the TRP domain, which comprises a helical segment roughly spanning from residues N990‐R1008 (in *hs*TRPM8, although this depends on the specific channel state, as evidenced from the available resolved structures), following transmembrane S6 helix. The role of the S6‐TRP linker has been analyzed by the group of A. Ferrer‐Montiel using a chimeric approach, replacing regions of this linker by the corresponding ones in TRPV1. This study reveals that the 980–985 chimera (^980^GETVNK^985^) leads to constitutively active channels, founding a pivotal role for *rn*TRPM8 Y981 residue.^85^ This publication, before the resolution of the 3D TRPM8 structures, includes residues 980–985 within the TRP domain, while after the resolution of the cryo‐EM structure, it was observed that this segment is within the S6 (structure of PDB code 6O77).^58^ TRPM8 mutants **Y981E** (containing the equivalent residue in TRPV1 channel) and **Y981K** render constitutively active channels, with the former leading to an increase in cell dead and the latter being toxic. Interestingly, the incorporation of further mutations to the 980–985 chimera in the adjacent segment 986–990, again replacing amino acids by the corresponding residues in TRPV1, restored both voltage‐dependent and menthol sensitivity to the chimeric channel. Interestingly, **V986I** prevented the constitutive activity, and a similar behavior was observed upon substitution by Ala, Leu and Phe, although there were differences, for example, in menthol sensitivity, depending on the amino acid volume. These authors suggested that Y981 and V986 participated in defining the energy of channel gating. Other mutations able to restore the regulated gating are **N989E** and **N990S**. On the other hand, mutated **Y981L** channel showed responses to voltage and menthol, although a decrease in menthol efficacy was observed, whereas, unpredictably, analog **Y981F** TRPM8 did not show membrane depolarization. In addition, studies on the mutation of V986 in WT *rn*TRPM8, within the S6‐TRP linker, through replacement by Ala, Gly and Phe, led to nonfunctional channels, whereas **V986L** mutant rendered a displacement of G–V curve to stronger depolarized potentials, and **V986I** had similar voltage sensitivity as the WT. The author suggested an allosteric contribution of V986 to channel activation. On the whole, these data are pointing to a relevant role of the S6, and S6‐TRP linker in channel gating.

Another important residue within the TRP domain was identified by Bandell et al.,^68^ who reported that alterations in the L1009 residue can lead to channel disfunctions. In Ca^2+^ imaging studies, the **L1009R**‐*mm*TRPM8 mutant showed a loss in menthol sensitivity compared to WT. On the contrary, voltage and PIP_2_ sensitivities were unaffected. Furthermore, high concentrations of menthol could potentiate the effect of cold temperatures. Icilin, however, did not elicit responses nor potentiated cold responses in this mutant. Interestingly, substitutions by other residues, as in **L1009A** and **L1009P** mutants (Pro residue is found at this position in other TRPM channels) exhibited a similar response to menthol as WT‐*mm*TRPM8, suggesting that Leu was not a key residue, and that the effect of **L1009R** mutant might be attributed to a disruption of the hydrophobic environment required for menthol binding. Shortly after this publication, in radiolabel‐binding studies with [^3^H]menthol the group of T. Voets observed that menthol binding to **L1009R** mutant was similar to WT *hs*TRPM8, which suggest that the mutation is affecting menthol efficacy.^73^ On the other hand, Bandell et al.^68^ described that the **Y1005F**‐*mm*TRPM8 variant showed activation by high menthol concentrations and cold (although reduced when compared to WT‐*mm*TRPM8), besides, voltage and PIP_2_ sensitivities were as in the WT‐channel. At this position, the mutation to alanine (**Y1005A**) led to further reduced menthol responses, which supported the hypothesis that both the aromatic ring and the hydroxyl group from Y1005 are involved in menthol binding, similarly to what has been found for Y745. In the cryo‐EM structure of the of TRPM8/menthol analog WS‐12 complex, PDB ID 6NR2, this Tyr (Y1004 in *fa*TRPM8) is within the binding pocket.^13^ In the TRP domain, Bandell et al.^68^ also analyzed substitutions of L1009, N1010, and I1011, leading to the triple mutant ^**1009**^ **PAA** ^**1011**^‐*mm*TRPM8, which display altered stereoselectivity to different menthol isomers, apparently as a result of diminished efficacy for some of them, while icilin could only induce slight responses at high concentrations, although this mutant maintained icilin‐induced cold potentiation.

The TRP domain has also been found to be important for PIP_2_ regulation. In 2005, Rohács et al.^12^ established that PIP_2_ is not only necessary for TRPM8 activation by other stimuli, but also sufficient, at high concentrations, to elicit currents by itself. Based on the hypothesis that PIP_2_ might interact with the TRP domain, these authors examined the behavior of TRPM8 mutants **K995Q**, **R998Q**, and **R1008Q** in response to PIP_2_. All of them showed reduced sensitivity to PIP_2_, and higher susceptibility to PIP_2_ depletion. Depending upon the mutations, the effect might vary, with the greater EC_50_ rightward shift for **R1008Q**. These data are indicating that PIP_2_ might interact with the TRP domain. In agreement, cryo‐EM structures have shown the implication of R998 in the interaction with PIP_2_ (R997 in *fa*TRPM8).^13^ Additionally, a reduced sensitivity to menthol was detected for the **R1008Q** mutant, and this mutant, together with the **R998Q** variant, was insensitive to icilin.

It is worth mentioning that mutants **K995Q** and **R1008Q** have been explored by Zhang et al.^104^ in the investigation on the mechanisms involved in the inhibition of *hs*TRPM8 channels by pro‐inflammatory mediators, such as bradykinin or histamine, which is mediated by the Guanine nucleotide‐binding q alpha subunit (Gα_q_). G_q_‐coupled GPCRs can activate PLCβ, leading to PIP_2_ hydrolysis, which in turn can inhibit TRPM8. However, U73122, a PLC inhibitor, does not prevent the inhibition of TRPM8 currents due to bradykinin or histamine. Besides, histamine was able to inhibit these two PIP_2_ insensitive mutants (**K995Q** and **R1008Q**). These data indicate that the inactivation was not via PIP_2_ hydrolysis. A new mechanism is proposed for this inhibition based on a direct binding of Gαq to TRPM8, and even a crosstalk with bradykinin B2R receptor, which supposedly binds by the N‐terminal domain of the channel.^105^

The cryo‐EM structures showed that pmTRPM8 R998 residue (R1008 in *hs*TRPM8) is involved in a salt bridge with Q776 (S2) in the AMTB bound structure, PDB ID 6O6R, whereas this interaction is missed in the structure of the Ca^2+^ bound desensitized state, PDB ID 6O77, allowing the TRP domain to adopt the desensitized conformation.^58^ At the C‐terminal domain, although the mutagenesis studies have focused mainly on the TRP domain, there are a couple of studies in other regions. Thus, Morgan et al. studied the double mutant **F1045A** + **K1046G**, which showed sensitivity to both agonists and antagonists. In fact, menthol or WS‐12 treatment led to transient intracellular Ca^2+^ elevation, which was blocked by the antagonist AMTB.^88^ On the other hand, the possibility of PIP_2_ interacting with TRPM8 C‐terminus has been explored by Rohács, analyzing a positive clusters spanning from residues 1026 to 1036, and particularly variants **K1026Q**, **K1027Q**, **K1030Q**, **K1034Q**, and **K1036Q**.^12^ However, **K1027Q** resulted in a nonfunctional channel, while the other four variants did not show any decrease in current amplitude, thus being unlikely to affect PIP_2_ binding.

## 3 CONCLUSIONS AND OUTLOOK

TRPM8 channels have been implicated in a broad range of pathologies, among them different pain types, including migraine, dry eye disease, inflammatory processes, and cancer. Therefore, this channel is being pursued as a valuable therapeutic target, and in fact different compounds have been developed for its modulation. It is expected that the number of TRPM8 modulators could increase in the near future, since their design can be guided by both mutagenesis and structural data.

Structural and mutagenesis studies are helping to unravel the molecular basis behind the response to thermal or chemical stimuli of TRPM8 channels. Thus, this has aided in the identification of residues that are involved in mediating the response to a particular stimulus, as G805 in *hs*TRPM8, corresponding to Ala in the avian channels,^77^ which are insensitive to icilin but maintain WT responses to menthol. Some residues have a pivotal role for the interaction with different ligands, as Y745, which upon mutation to His led to a channel insensitive to agonists like menthol or icilin, partial agonists, as praziquantel, or antagonists, as SKF96365, whereas it shows WT response to cold.^68^, ^70^, ^71^ On the contrary, H845A or R862A display similar sensitivity to menthol as the WT, but a reduced response to cold.^73^ Hence, these studies have shown that a particular chimera or mutant can selectively modify the current evoked by a single stimulus, while maintaining its sensitivity against others.

The recognition that a chimera or mutant can modify the response to a single stimulus, could be of special relevance to direct the selection of candidates for clinical studies with less side‐effects. For example, clinical trials with the TRPM8 antagonist PF‐05105679 showed hot sensation as an insuperable adverse effect (together with short haft‐live) which preclude further clinical studies.^106^

More recently, comparison of sequences, chimeras, and mutagenesis, are shedding light regarding the adaptation of species to live in different environments, from the cold polar areas to the hot deserts. These are of interest to get insights into the mechanisms that have driven the adaptation of species to different ambient conditions during evolution, and also provide evidence of residues involved in cold response.

In the last years, the field of TRPM8 has also experienced a great advance with the resolution of its 3D cryo‐EM structures, first the apo structure of the channel, and then in complex with different ligands, both agonist and antagonist. The analysis of these structures, together with the mutagenesis data, will foster the computer‐assisted rational design of novel chemotypes able to interact with particular residues promoting a biological effect, while keeping WT‐like response to thermal stimuli.

On the next years, the interplay of structural and mutagenesis studies, together with structure–activity relationships of known ligands, will contribute to a better understanding of these channels. Therefore, this might lead to the development of novel modulators able to fine‐tune a particular TRPM8 response, which will be of great interest for the devolvement of novel TRPM8‐based therapies with reduced adverse effects.

## Supporting information

Supporting information.

## Data Availability

Data sharing not applicable to this article as no new data were generated or analyzed during the current study.
