# Supplementary material for: Mutations of TRPM8 channels: Unraveling the molecular basis of activation by cold and ligands
Source: Med Res Rev. 2022 Aug 17;42(6):2168–203. doi: 10.1002/med.21920 (PMC9805079; doi:10.1002/med.21920)
Supplement: Supplementary file 1 — Supporting information. [file MED-42-2168-s001.docx]

**Mutations of TRPM8 channels: unraveling the molecular basis of activation by cold and ligands**

Alejandro Plaza-Cayón, Rosario González-Muñiz, Mercedes Martín-Martínez

Supporting Information

Figure S1. Structure of the cooling agent 10 and a naphthyl antagonist.


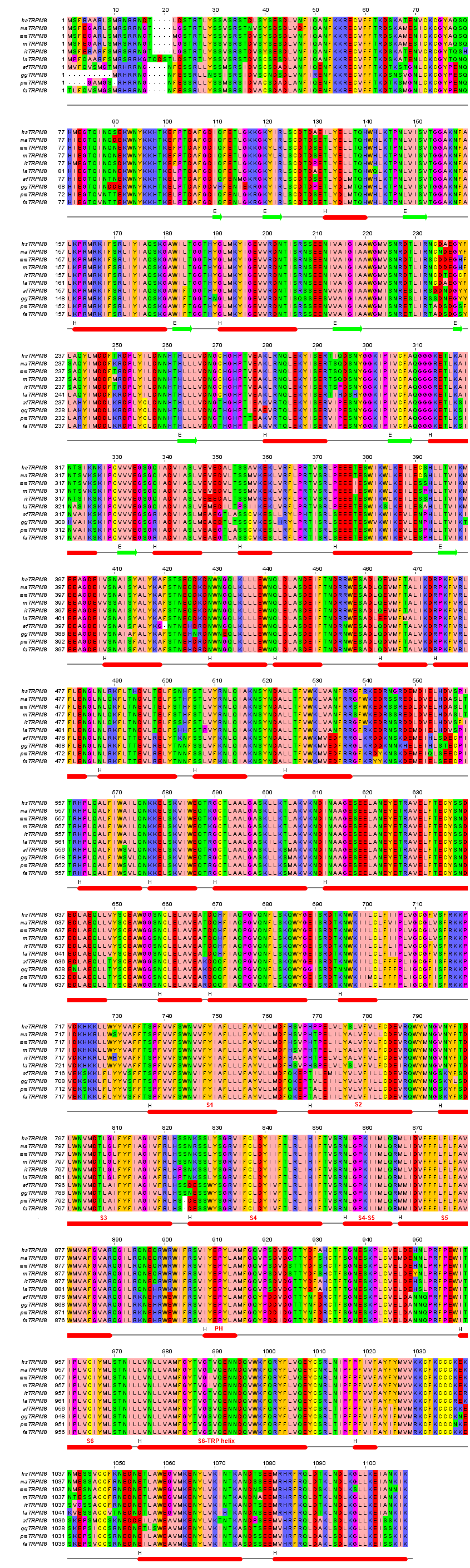


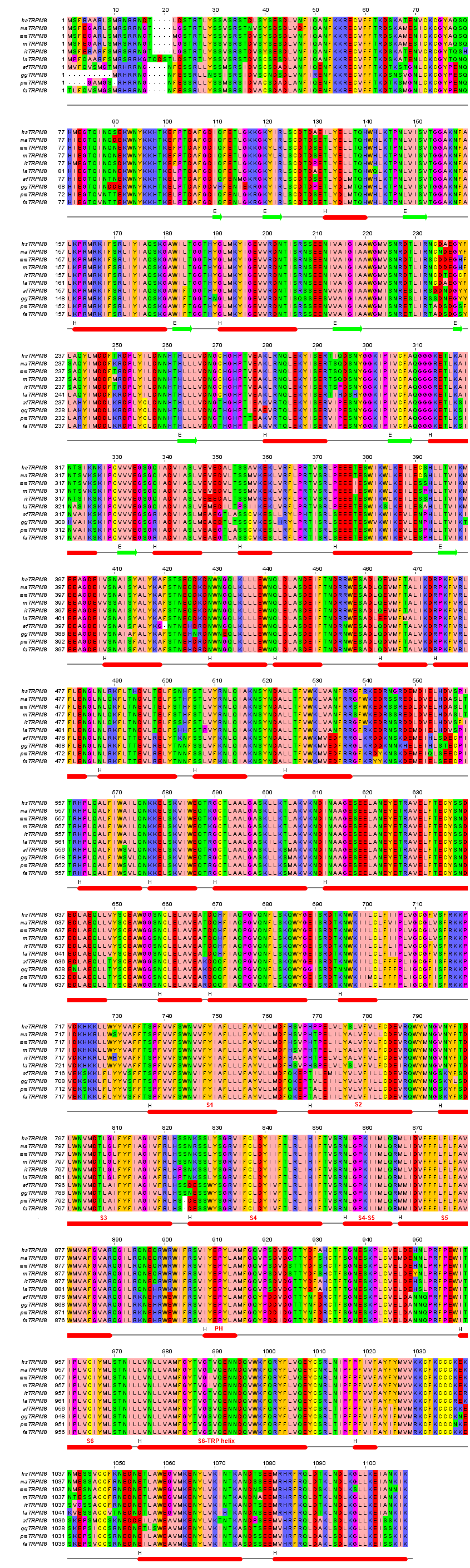


**Figure S2.** TRPM8 protein sequence alignment of the species discussed. ***hs*TRPM8**: human (*Homo sapiens*) TRPM8, ***ma*TRPM8**: Syrian hamster (*Mesocricetus auratus*) TRPM8, ***mm*TRPM8**: mouse (*Mus musculus*) TRPM8, ***rn*TRPM8**: rat (*Rattus norvegicus*) TRPM8, ***it*TRPM8**: thirteen-lined ground squirrels (*Ictidomys tridecemlineatus*) TRPM8, ***la*TRPM8**: african elephant (*Loxodonta africana*) TRPM8, ***af*TRPM8**: emperor penguin (*Aptenodytes forsteri*) TRPM8, ***gg*TRPM8**: chicken (*Gallus gallus*) TRPM8, ***pm*TRPM8**: great tit (*Parus major*) TRPM8, ***fa*TRPM8**: collared flycatcher (*Ficedula albicollis*) TRPM8. The secondary structure, depicting β-sheets as green arrows and α-helices as red bars, was retrieved from the pmTRPM8 cryo-electronic microscopy structure PDB ID 6O77.
